# Supplementary material for: Genetic Polymorphisms (ApaI, FokI, BsmI, and TaqI) of the Vitamin D Receptor (VDR) Influence the Natural History and Phenotype of Crohn’s Disease
Source: Int J Mol Sci. 2025 Feb 21;26(5):1848. doi: 10.3390/ijms26051848 (PMC11899612; doi:10.3390/ijms26051848)

SUPPLEMENTARY MATERIALS

Supplementary Table S1. Distribution of ApaI, BsmI, TaqI, and FokI polymorphisms in relation to epidemiological and clinical characteristics of IBD patients

		Family History of IBD			Presence of extra-intestinal manifestations			Coexisting auto-immune diseases		
		YES	NO	Sig	YES	NO	sig	YES	NO	sig
ApaI	aa	1	26	0.051	8	19	0.85	2	25	0.828
	Aa	1	61		17	48		6	59	
	AA	6	43		16	36		6	46	
BsmI	bb	2	50	0.681	16	38	0.582	6	48	0.904
	Bb	5	61		21	48		6	63	
	BB	1	19		4	17		2	19	
TaqI	tt	4	47	0.48	17	35	0.646	7	45	0.523
	Tt	2	61		18	48		5	61	
	TT	2	22		6	20		2	24	
FokI	ff	0	16	0.561	2	14	0.236	0	16	0.26
	Ff	4	53		20	39		5	54	
	FF	4	61		19	50		9	60	

Supplementary Table S2. Haplotype profiling and association with IBD-related hospitalization in Crohn's disease patients.

Haplotype	Case(freq)	Control(freq)	χ^2	Fisher's p	Pearson's p	OR [95% CI]	Adjusted p-value
abtF	16(0.181)	23(0.359)	6.124	**0.015***	**0.013***	0.396 [0.188~0.833]	**0.029***
abtf	8(0.09)	10(0.156)	1.515	0.309	0.218	0.54 [0.2~1.455]	0.3
ABtF	3(0.034)	2(0.031)	0.009	1	0.922	1.094 [0.177~6.745]	0.922
ABTF	17(0.193)	6(0.093)	2.852	0.11	0.091	2.314 [0.857~6.249]	0.167
ABTf	21(0.238)	12(0.187)	0.57	0.551	0.45	1.358 [0.612~3.011]	0.55
AbtF	12(0.136)	8(0.125)	0.041	1	0.837	1.105 [0.423~2.883]	0.921
AbTF	6(0.068)	1(0.015)	2.329	0.239	0.126	4.609 [0.541~39.272]	0.199

Global result: Total control=32, total case=44. Global Chi^2 is 10.953, Pearson's p is 0.089.

Supplementary Table S3. Haplotype profiling and association with IBD-related surgery in Crohn's disease patients

Haplotype	Case(freq)	Control(freq)	χ^2	Fisher's p	Pearson's p	OR [95% CI]	Adjusted p-value
abtF	7(0.125)	32(0.333)	8.047	**0.006***	**0.004***	0.285 [0.116~0.701]	**0.01***
abtf	5(0.089)	13(0.135)	0.72	0.447	0.395	0.625 [0.21~1.859]	0.469

ABtF	1(0.017)	4(0.041)	0.63	0.652	0.427	0.418 [0.045~3.837]	0.469
ABTF	13(0.232)	10(0.104)	4.51	0.058	**0.033***	2.6 [1.054~6.408]	0.061
ABTf	13(0.232)	20(0.208)	0.117	0.838	0.731	1.148 [0.52~2.536]	0.731
AbtF	10(0.178)	10(0.104)	1.713	0.218	0.19	1.869 [0.725~4.818]	0.299
AbTF	4(0.071)	3(0.031)	1.299	0.424	0.254	2.384 [0.513~11.067]	0.349

Total control=48, total case=28. Global Chi^2 is 13.917, Pearson's p is **0.03***.

Supplementary Figure S1. Triangular heatmap visualization of pairwise linkage disequilibrium between vitamin D receptor variants in Ulcerative Colitis (left) and Crohn's Disease (right) patients. Each square represents the D' value between pairs of polymorphisms.

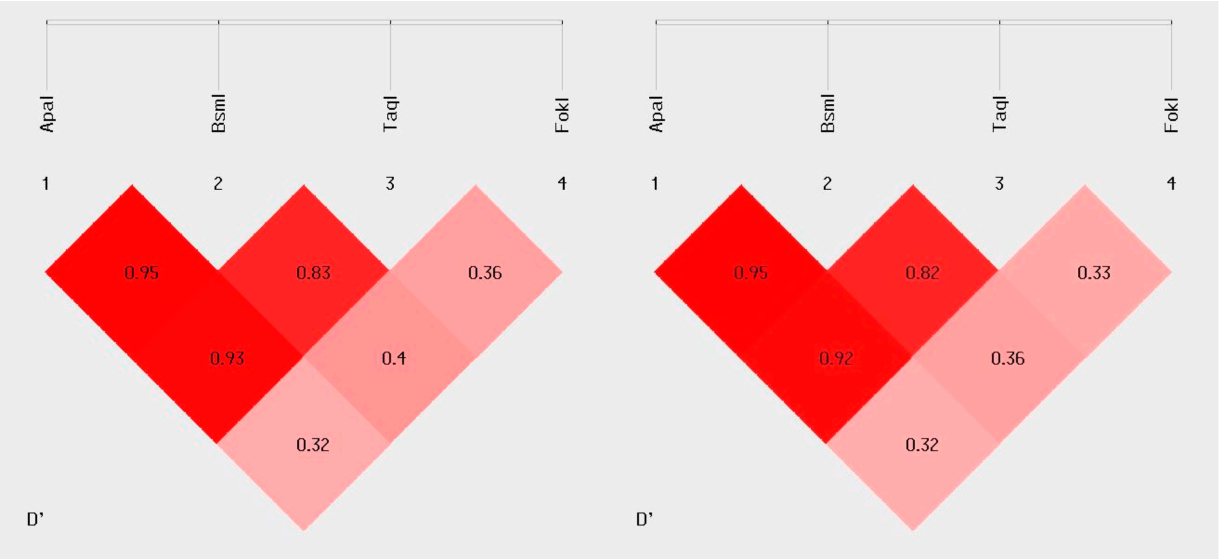

Supplement: Supplementary file 1 [file ijms-26-01848-s001.zip › ijms-3416777-supplementary.pdf]
